# Utilization of leadless pacemaker following orthotopic heart transplantation for complete heart block during index hospitalization

**DOI:** 10.1016/j.hrcr.2024.09.010

**Published:** 2024-09-18

**Authors:** Radha Gopalan, Hayley Mitchel, Ritika Gupta, Francisco Arabia, Praneeth Katrapati, Wilber Su

**Affiliations:** 1 Advanced Heart Failure and Transplant Department, University of Arizona College of Medicine – Phoenix, Phoenix AZ; 2 Midwestern University Arizona College of Osteopathic Medicine, Glendale, AZ; 3 Electrophysiology Department, University of Arizona College of Medicine – Phoenix, Phoenix AZ

**Keywords:** Leadless pacemaker, Pacemaker, Heart, Transplant, Heart block

**Key Teaching Points**

• Heart block is a frequent complication after heart transplantation. Up to 10% of patients will require permanent pacing after transplantation.
• Leadless pacemakers offer several benefits over traditional pacemakers, including smaller size, less invasive implantation procedure, and less risk of lead dislodgement.
• Graft conduction dysfunction will recover in a significant number of patients after heart transplantation. In this scenario, a leadless pacemaker can be easily removed via a minimally invasive catheter-based retrieval system.
• A limitation of current leadless pacemaker devices is the inability to upgrade to cardiac resynchronization therapy in the setting of pacemaker-induced ventricular dysfunction.

## Introduction

Permanent pacemaker (PPM) implantation is an established practice for treating conduction abnormalities after heart transplantation, and is reported in approximately 2%–10% of heart transplant patients.^1^, ^2^, ^3^, ^4^ Early (<30 days) postoperative conduction abnormalities are often the result of surgical trauma, ischemic injury due to prolonged ischemic time of the donor heart, nodal dysfunction from pretransplantation use of amiodarone, and/or acute rejection, and are more common after biatrial than bicaval technique. Complicating the decision to implant PPM after heart transplantation is the lower rate of permanent pacing dependency compared with the rate of initial nodal dysfunction in this population.^5^ Early postoperative conduction abnormalities typically resolve and can be treated with temporary pacing. Some patients, however, experience persistent and significant dysfunction requiring PPM implantation during the immediate postoperative period.

Complications from traditional PPM implantation in the general population are reported in the literature at rates as high as 20%.^6^, ^7^, ^8^ Complications include, but are not limited to, infection, pneumothorax, cardiac perforation, hematoma, tricuspid regurgitation, and lead dislodgement. Careful consideration is given when implanting a transvenous PPM after heart transplantation, due to the immunocompromised status of these patients placing them at high risk of developing infectious complications. Post-transplantation patients are especially vulnerable in the immediate (hours to weeks) postoperative period when the immune system is exceedingly depressed to prevent acute rejection of the graft. Infection-related mortality has been reported at higher rates in early postoperative PPM implantation compared with late PPM implantation.^9^^,^^10^

Leadless pacemakers (L-PMs) are an alternative to traditional PPMs for patients requiring single- or dual-chamber pacing, and their use aims to decrease the incidence of postimplantation complications. Here we present the first reported case of a patient implanted with an L-PM in the immediate postoperative period to treat postoperative atrioventricular block after heart transplantation.

## Case report

**Figure 1:**
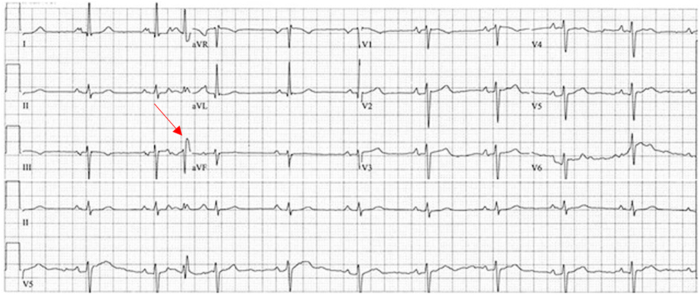
Electrocardiogram with right bundle branch block within premature atrial contraction.

**Figure 2:**
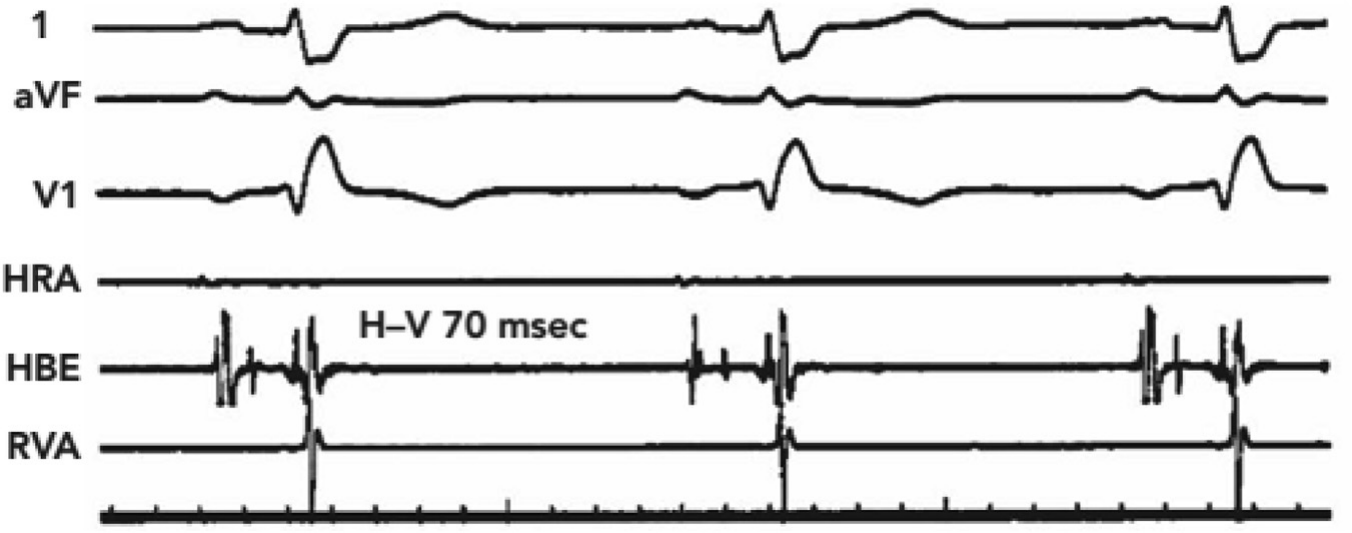
Electrophysiological study with prolonged HV interval of 70 milliseconds.

**Figure 3:**
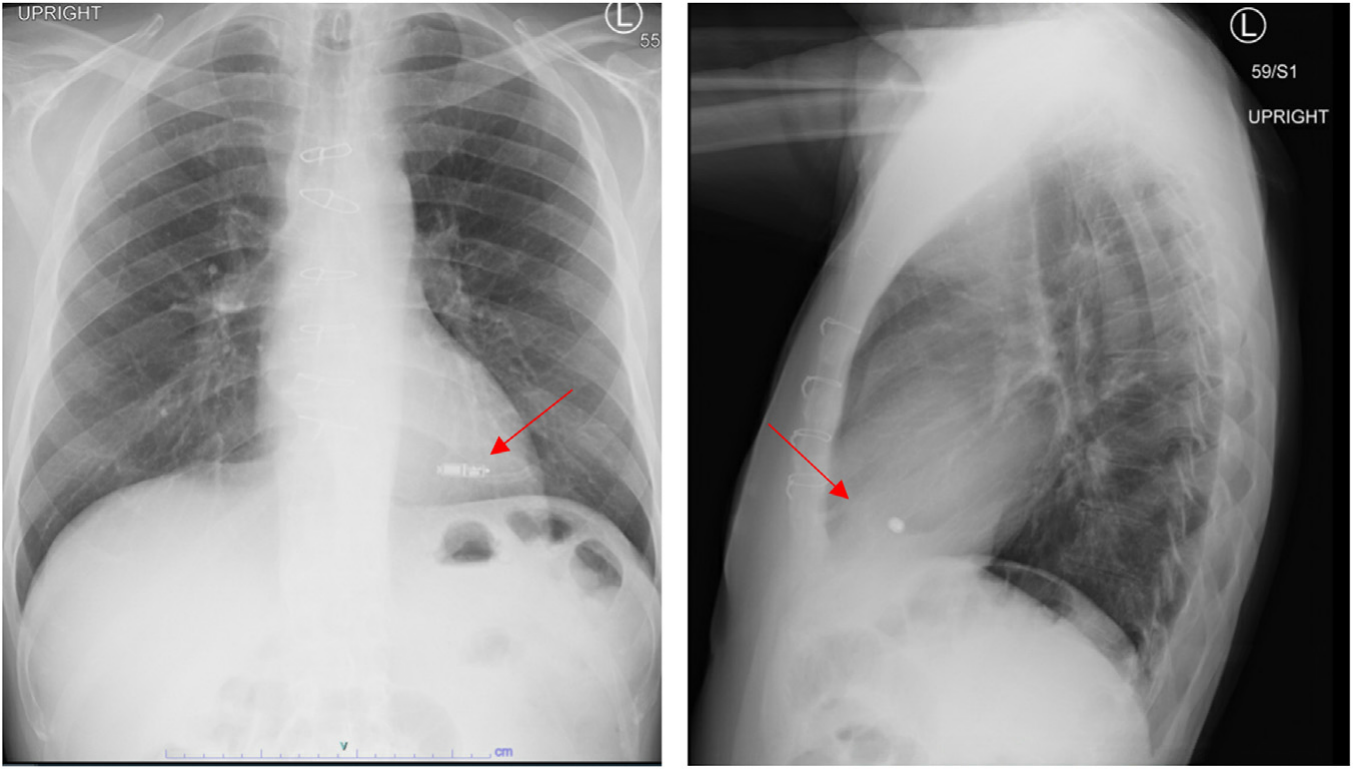
Chest posteroanterior and lateral showing Micra AV leadless pacemaker (Medtronic, Minneapolis, MN) in the right ventricular apex.

The patient was a 67-year-old man with a medical history significant for dilated nonischemic cardiomyopathy status post implantable cardioverter-defibrillator placement, transcatheter aortic valve implantation, and atrial fibrillation ablation. The patient underwent orthotopic heart transplantation via the bicaval technique as United Network for Organ Sharing status 2 with temporary epicardial pacing wires placed intraoperatively per standard protocol. Following surgery, the patient was noted to be in complete heart block without ventricular escape rhythm. On postoperative day 3, the patient sustained a loss of capture, resulting in 2 episodes of asystole with successful return of spontaneous circulation followed by surgical placement of 2 new epicardial wires. The patient continued to have labile atrioventricular conduction. Electrophysiological study conducted on postoperative day 21 showed baseline sinus rhythm with frequent premature atrial contractions and alternating bundle branch block (Figure 1). HV interval was 70 milliseconds (Figure 2). Due to persistent infra-Hisian disease, the patient underwent placement of a leadless Micra AV PPM (Medtronic, Minneapolis, MN) (Figure 3) set to VDD with lower rate set at 50 beats/min. There were no postoperative complications, and the patient was discharged home on postoperative day 37. One year after implantation, ventricular pacing by the device was activated <1% of the time and ventricular sensing was active 100% of the time (of note, the Micra LPM implanted in this case does not have true atrial sensing capabilities). The patient subsequently underwent post-transplantation surveillance endomyocardial biopsies (EMB) per institution protocol:

• Month 1: EMB weekly
• Months 2 and 3: EMB every 2 weeks
• Months 4–12: EMB monthly

At the time of this writing, no device-related complications have occurred since discharge.

## Discussion

The Micra AV L-PM is an alternative option to the traditional PPM implant in patients who require single-chamber ventricular pacing (since the time of this writing, several atrioventricular leadless pacemakers have been approved for patients requiring dual-chamber pacing). Short- and long-term complications with traditional PPMs are primarily associated with the subcutaneous pocket and transvenous lead. These factors are completely circumvented in the design of L-PMs, greatly reducing the overall risk of adverse events. The pulse generator and battery are contained within the device and affixed to the right ventricular apex. The device provides atrial-synchronous ventricular pacing via mechanical tracking of atrial contraction, as opposed to the electrical sensing of atrial contraction used in traditional dual-chamber pacemakers. Implantation uses a minimally invasive percutaneous catheter delivery system to fix the device within the right ventricular apex, which decreases the risk of infection and endocarditis. The risk of device dislodgment in leadless pacemakers has been reported at significantly lower rates compared with rates in traditional PPMs.^11^ This is especially crucial in heart transplant patients who undergo frequent EMBs, placing them at higher risk of lead dislodgement. In addition, although many patients will require initial pacemaker dependency after heart transplantation, conduction dysfunction recovers in a significant number of patients,^5^ as was observed in our patient. The design of L-PMs permits easy removal via a retrieval catheter, a less invasive technique than removal of traditional pacemakers. The device is also 93% smaller than traditional PPMs, maximizing patient comfort and minimizing cosmetic concerns.

In the past decade, leadless pacemakers have been found to be as effective and safer compared with traditional PPMs in patients who have received a pacemaker >1 year post transplantation. Araj and colleagues^12^ were the first to report a case of an L-PM implant in a heart transplant patient in 2019, which was implanted 5 years post transplantation.^12^ In a multicenter longitudinal observation study, 7 patients that required permanent pacing were implanted with the Medtronic Micra L-PM >1 year post heart transplantation.^13^ In both studies, all but 1 patient had no complications 1 year after implantation, and the efficacy of the device had no statistically significant difference compared with conventional pacemakers.

Although L-PMs have shown decreased complications compared with traditional PPMs, a major limitation to their use is the inability to upgrade to cardiac resynchronization therapy (CRT). An estimated 10%–15% of patients develop pacing-induced cardiomyopathy and a decrease in left ventricular ejection fraction ≥10% after device implant.^14^ Traditional PPMs can be upgraded to deliver CRT therapy in these cases to minimize ventricular dyssynchrony. L-PMs do not currently have CRT capabilities, and thus patients may require removal of the L-PM to upgrade to CRT via a traditional PPM or may result in persistent ventricular dysfunction/heart failure.^15^ CRT pacing capabilities with L-PMs are, however, currently under investigation, at the time of this writing, with promising results in recent clinical trials.^16^ It is also worth noting that pacing-induced cardiomyopathy is reported at significantly lower rates with L-PMs compared with traditional PPMs (3% vs 13.7%, respectively), and there is some evidence to suggest that septal implantation is associated with lower rates of pacing-induced cardiomyopathy compared with nonseptal implantation.^17^

## Conclusion

We are the first to report a case where the Micra pacemaker was placed in a patient status post heart implantation in the immediate postoperative period with no complications to date. This case provides evidence for the feasibility, efficacy, and advantages of leadless PPM use in the immediate postoperative period after orthotopic heart transplantation. As a single-patient case report, this study is limited by its inherently small sample size, and further studies are needed to give strength to the feasibility of using L-PM in the immediate postoperative period after transplantation.

## Disclosures

The authors have no conflicts of interest to disclose.
